# 188 Impact of Stewardship Reimplementation on Inappropriate Antibiotic Prescribing for Acute Respiratory Infections in Urgent Care

**DOI:** 10.1017/ash.2026.10580

**Published:** 2026-06-23

**Authors:** Danielle Casaus, Rachel Hish, Derek Forster, Wael Ibrahim, Joel Howard, Katie Wallace, Randal de Souza, Armaghan-e-Rehman Mansoor

**Affiliations:** 1 University of Kentucky Healthcare; 2 UK HealthCare; 3 University of Kentucky School of Medicine; 4 University of Kentucky, College of Medicine; 5 University of Kentucky

## Abstract

**Background:** Community-acquired pneumonia (CAP) is most commonly caused by Streptococcus pneumoniae, Haemophilus influenzae, Moraxella catarrhalis or atypical bacteria. Legionella pneumonia is most commonly associated with contaminated man-made water systems. The 2019 CAP guidelines endorsed by the American Thoracic Society and Infectious Diseases Society of America are the only national guidelines that recommend the Streptococcus pneumoniae urinary antigen test (UAT) in patients presenting with severe CAP, and the Legionella UAT in patients presenting with either severe CAP or with recent travel to an area associated with a Legionella outbreak. Since the publication of this guideline, several publications have questioned the utility of these UATs in clinical practice. **Methods:** We conducted a retrospective review of Streptococcus pneumoniae and Legionella UATs ordered for inpatient adult patients at the University of Kentucky Healthcare during the calendar year 2024. Chart review was conducted for all patients with a positive UAT to determine indication and appropriateness of test ordering, and details of antimicrobial prescription and duration. **Results:** Of the 6,421 orders, only 168 (2.8%) were positive – 153 Streptococcus pneumoniae UAT and 15 Legionella UAT. Patients with a positive test were reviewed to determine diagnostic indication for the test as well as to evaluate antibiotic de-escalation practices. Of the positive UATs, 37.5% of the orders were obtained in patients who did not have CAP. Most common indications were VAP, HAP, aspiration pneumonia, acute respiratory failure, sepsis, COPD exacerbations, and CHF exacerbations. None of the patients with a positive Streptococcus pneumoniae UAT were narrowed to amoxicillin to target the pathogen. Most commonly, patients were narrowed from cefepime to ceftriaxone or atypical coverage was discontinued. For patients with Legionella pneumonia, macrolide therapy was extended beyond the typical three-day regimen used in CAP or changed to a respiratory fluoroquinolone. **Conclusion:** Based on our review, we are recommending removing the Streptococcus pneumoniae UAT from our institution’s formulary and optimizing the Legionella UAT order by limiting use to patients with risk factors. This proposal will result in significant cost savings as our enterprise spent $164,211 on UATs that did not significantly influence care for most patients.

